# Longitudinal changes in rich club organization and cognition in cerebral small vessel disease

**DOI:** 10.1016/j.nicl.2019.102048

**Published:** 2019-10-22

**Authors:** Esther M.C. van Leijsen, Ingeborg W.M. van Uden, Mayra I. Bergkamp, Helena M. van der Holst, David G. Norris, Jurgen A.H.R. Claassen, Roy P.C. Kessels, Frank-Erik de Leeuw, Anil M. Tuladhar

**Affiliations:** a Department of Neurology*,* Donders Institute for Brain*,* Cognition and Behaviour*,* Donders Center for Medical Neuroscience*,* Radboud University Medical Center*,* Nijmegen*,* the Netherlands; b Department of Neurology, Jeroen Bosch Ziekenhuis, ’s-Hertogenbosch, the Netherlands; c Radboud University, Donders Institute for Brain, Cognition and Behaviour, Centre for Cognitive Neuroimaging, Nijmegen, the Netherlands; d Erwin L. Hahn Institute for Magnetic Resonance Imaging, University of Duisburg-Essen, Essen, Germany; e Department of Geriatric Medicine, Donders Institute for Brain, Cognition and Behaviour, Donders Center for Medical Neuroscience, Radboud University Medical Center, Radboud Alzheimer Centre, Nijmegen, the Netherlands; f Department of Medical Psychology, Radboud university medical centre, Radboud Alzheimer Centre, Nijmegen, the Netherlands; g Radboud University, Donders Institute for Brain, Cognition and Behaviour, Centre for Cognition, Nijmegen, the Netherlands

**Keywords:** Cerebral small vessel disease, Structural neuroimaging, Diffusion tensor imaging, Cognitive decline, Dementia, Rich club organization

## Abstract

Cerebral small vessel disease (SVD) is considered the most important vascular contributor to the development of cognitive impairment and dementia. There is increasing awareness that SVD exerts its clinical effects by disrupting white matter connections, predominantly disrupting connections between rich club nodes, a set of highly connected and interconnected regions. Here we examined the progression of disturbances in rich club organization in older adults with SVD and their associations with conventional SVD markers and cognitive decline. We additionally investigated associations of baseline network measures with dementia. In 270 participants of the RUN DMC study, we performed diffusion tensor imaging (DTI) and cognitive assessments longitudinally. Rich club organization was examined in structural networks derived from DTI followed by deterministic tractography. Global efficiency (*p*<0.05) and strength of rich club connections (*p*<0.001) declined during follow-up. Decline in strength of peripheral connections was associated with a decline in overall cognition (β=0.164; *p*<0.01), psychomotor speed (β=0.151; *p*<0.05) and executive function (β=0.117; *p*<0.05). Baseline network measures were reduced in participants with dementia, and the association between WMH and dementia was causally mediated by global efficiency (*p* = =0.037) and peripheral connection strength (*p* = =0.040). SVD-related disturbances in rich club organization progressed over time, predominantly in participants with severe SVD. In this study, we found no specific role of rich club connectivity disruption in causing cognitive decline or dementia. The effect of WMH on dementia was mediated by global network efficiency and the strength of peripheral connections, suggesting an important role for network disruption in causing cognitive decline and dementia in older adults with SVD.

## 1 Introduction

Cerebral small vessel disease (SVD) is considered the most important vascular contributor to the development of cognitive impairment and dementia (Banerjee et al., 2016; Gorelick et al., 2011; Prins and Scheltens, 2015), but exactly *how* SVD results in cognitive decline or dementia is hitherto incompletely understood (Patel and Markus, 2011; Wardlaw et al., 2013). There is increasing awareness that SVD exerts its clinical effects by disrupting white matter connections (Lawrence et al., 2014; O'Sullivan et al., 2005, 2001; Tuladhar et al., 2016a).

Several cross-sectional studies in patients with SVD have shown that reduced structural network integrity, reflected by decreased global efficiency, was related to increased cognitive impairment (Lawrence et al., 2014; Reijmer et al., 2015; Tuladhar et al., 2015, 2016a) and to an increased risk of future dementia (Tuladhar et al., 2016b). Reduced connectivity was predominantly observed for connections between so-called rich club nodes (Tuladhar et al., 2017) – nodes that are both highly connected to the network and highly interconnected with each other (van den Heuvel et al., 2012; van den Heuvel and Sporns, 2011). Moreover, rich club connectivity strength mediated the association of WMH with processing speed and executive functioning, such that higher rich club connectivity strength was associated with better cognitive performance (Tuladhar et al., 2017). However, how SVD-related disturbances in rich club organization progress over time and how this relates to subsequent cognitive decline is unknown.

We hypothesize that disturbances in the rich organization progress over time in parallel with disease progression and that this is associated with cognitive decline and dementia. We therefore longitudinally examined the progression of disturbances in rich club organization in 270 participants with SVD and their associations with conventional SVD markers and cognitive decline. We additionally investigated associations of baseline network measures with dementia.

## 2 Material and methods

### 2.1 Study population

This study was part of the Radboud University Nijmegen Diffusion tensor and Magnetic resonance imaging Cohort (RUN DMC) study, a prospective cohort study of 503 older adults with SVD that investigates risk factors and clinical consequences of SVD. The detailed study protocol has been published previously (van Norden et al., 2011). In the present study, we only used data available from the first (2011) and second (2015) follow-up assessments and excluded data from the baseline (2006) assessment due to slight changes in scanner protocol between 2006 and 2011. In the rest of this article, we will refer to the 2011 assessment as ‘baseline’ and to the 2015 assessment as ‘follow-up’. Of the 503 participants (during the 2006 examination), 329 participants were available for baseline (2011) analyses. In addition, 281 participants underwent repeated neuroimaging assessments (van Leijsen et al., 2017), 11 of whom were excluded because of neuroimaging artefacts, yielding 270 participants for the longitudinal analyses.

### 2.2 Standard protocol approvals, registrations, and patient consents

The Medical Review Ethics Committee region Arnhem-Nijmegen approved the study and all participants gave written informed consent.

### 2.3 Cognitive assessment

Cognitive performance was measured using an extensive neuropsychological test battery during all waves of data collection, as has been described previously (van Uden et al., 2015a). Raw scores of all time-points were transformed into z-scores based on the mean and standard deviation (SD) of the baseline study population. We calculated Speed–Accuracy Trade-Off (SAT) scores where appropriate. Cognitive decline over time was calculated for each participant individually, by subtracting baseline scores from the follow-up scores.

We calculated a compound score for global cognitive function (cognitive index) as well as for three cognitive domains: memory, psychomotor speed and executive function. For the cognitive index, we calculated the mean of the z-scores of all tests from the neuropsychological test battery. To measure memory, we used the immediate and delayed recall of the Rey Auditory Verbal Learning Test (RAVLT) (Van der Elst et al., 2005) and the Rey Complex Figure Task (RCFT) (Caffarra et al., 2002), as well as Speed–Accuracy Trade-Off (SAT) scores of the 2- and 3-letter subtasks of the Paper-Pencil Memory Scanning Task (PPMST) (Van Der Elst et al., 2007). Psychomotor speed was calculated as the mean of the z-scores of the 1-letter subtask of the PPMST, the reading and color naming tasks of an adapted version of the Stroop Test (Van der Elst et al., 2006c) and the Symbol Digit Substitution Task (SDST) (van der Elst et al., 2006a). For executive function, we calculated the interference score of the Stroop Test by dividing SAT-scores of the color-word task by the mean SAT-scores of the reading and color naming tasks of the Stroop Test (Houx et al., 1993), the verbal fluency task (Van der Elst et al., 2006b) and SAT-scores of the Verbal Series Attention Test (VSAT) (Mahurin and Cooke, 1996). To account for possible material-specific practice effects, parallel versions of the RAVLT, RCFT and verbal fluency test were used for the follow-up assessment.

### 2.4 Dementia diagnosis

Dementia case finding was extensively described previously (van Uden et al., 2015b). In short, dementia was diagnosed after outpatient evaluation of the individual patient findings at the Radboud Alzheimer Center memory clinic, or by a consensus diagnosis by a panel consisting of a neurologist, clinical neuropsychologist and a geriatrician with expertise in dementia, who reviewed all available cognitive assessments and medical records. The diagnosis of dementia was based on the DSM-IV-TR criteria (American Psychiatric Association, 2000). In total, 23 out of 329 participants were diagnosed with dementia at follow-up.

### 2.5 Vascular risk factors

We recorded the presence of hypertension, smoking, alcohol use, diabetes and hypercholesterolemia at baseline by standardized questionnaires and physical examinations, as described previously (van Norden et al., 2011). We defined hypertension as the use of antihypertensive agents and/or systolic blood pressure greater than or equal to 140 mm Hg and/or diastolic blood pressure greater than or equal to 90 mm Hg (van Norden et al., 2011).

### 2.6 MRI acquisition

MR images were acquired at two time points (2011 and 2015) on the same 1.5-Tesla Siemens Magnetom Avanto scanner and included the following whole brain scans: T1-weighted 3D MPRAGE imaging (isotropic voxel size 1.0 mm^3^), a FLAIR sequence (voxel size 0.5 × 0.5 × 2.5 mm; interslice gap 0.5 mm) and a DTI sequence (isotropic voxel size 2.5 mm^3^, 8 unweighted scans, 60 diffusion weighted scans at *b* = =900 s/mm^2^). Full acquisition details have been described previously (van Leijsen et al., 2017; van Norden et al., 2011).

### 2.7 Conventional markers for SVD and brain volumetry

We calculated grey matter (GM), white matter (WM) and CSF volumes using SPM12 (https://www.fil.ion.ucl.ac.uk/spm/) unified segmentation routines on the T1 MPRAGE images, which were first corrected for the presence of WMH as described in (van Leijsen et al., 2017). All segmentations were visually checked for artefacts and segmentation errors and excluded from analyses when necessary.

White matter hyperintensities (WMH) were segmented semi-automatically using FLAIR and T1 sequences (Ghafoorian et al., 2016). All segmentations were visually checked for segmentation errors by one trained rater, blinded for clinical data. WMH volumes were calculated in ml, corrected for interscan differences in intracranial volume (ICV) and normalized to baseline ICV (van Leijsen et al., 2017).

### 2.8 DTI preprocessing

All diffusion weighted images were denoised using a Local Principal Component Analyses filter (Manjon et al., 2013), and corrected for cardiac, head motion, and eddy current artefacts simultaneously using the ‘PATCH’ algorithm (Zwiers, 2010), as described previously (Tuladhar et al., 2017; van Uden et al., 2016). Diffusion tensor and scalar parameters were calculated using DTIFIT from FSL's FDT toolbox. Whole-brain deterministic tractography has been described previously (Lawrence et al., 2014). Streamlines were terminated at an angle, exceeding 40° between principal eigenvectors, or FA<0.2.

### 2.9 Network nodes and edges

Brain regions were parcellated in each participant using the Automatic Anatomical Label (AAL) template (Tzourio-Mazoyer et al., 2002) into 90 regions, excluding the cerebellar regions. For each participant, T1-weighted images were first linearly registered to the b0-image using FMRIB's Linear Image Registration Tool (FLIRT), part of FSL. The T1-weighted images were then non-linearly registered to Montreal Neurological Institute (MNI) 152 template using ANTs. Linear and non-linear transformations were finally combined to register the AAL template to each participant's diffusion space.

Two regions were considered connected if the endpoints of a tractography streamline were located within the pair of brain regions. Connection strengths were estimated based on a modified method of Hagmann and colleagues (Hagmann et al., 2007; Lawrence et al., 2014) and calculated as the sum of the inverse of the streamlines length, including a scaling factor to correct for the number of seeds per squared millimeter. Strengths were calculated for each connection from the number of streamlines, with adjustments to correct for distance traveled and the seeding scheme. For each streamline, the inverse length was calculated and summed. This adjustment is needed to correct for linear bias towards the longer fiber by the tractography technique (Hagmann et al., 2007). Weighted edges were thresholded at 1, to reduce noise-related false-positive connections. This resulted in individual weighted connectivity matrices.

### 2.10 Network measures

Graph theoretical measures were calculated from the structural network using the Brain Connectivity Toolbox (Rubinov and Sporns, 2010) (https://sites.google.com/site/bctnet/). These measures included: (1) node degree, representing the number of connections of a node; (2) network density, defined as the ratio between the number of connections present and the number of total possible connections in a network; (3) total network strength, computed as the sum of all connection strengths in a network; (4) global efficiency, expressed as the average inverse of the shortest path length between two nodes.

### 2.11 Rich club measures

**Fig. 1:**
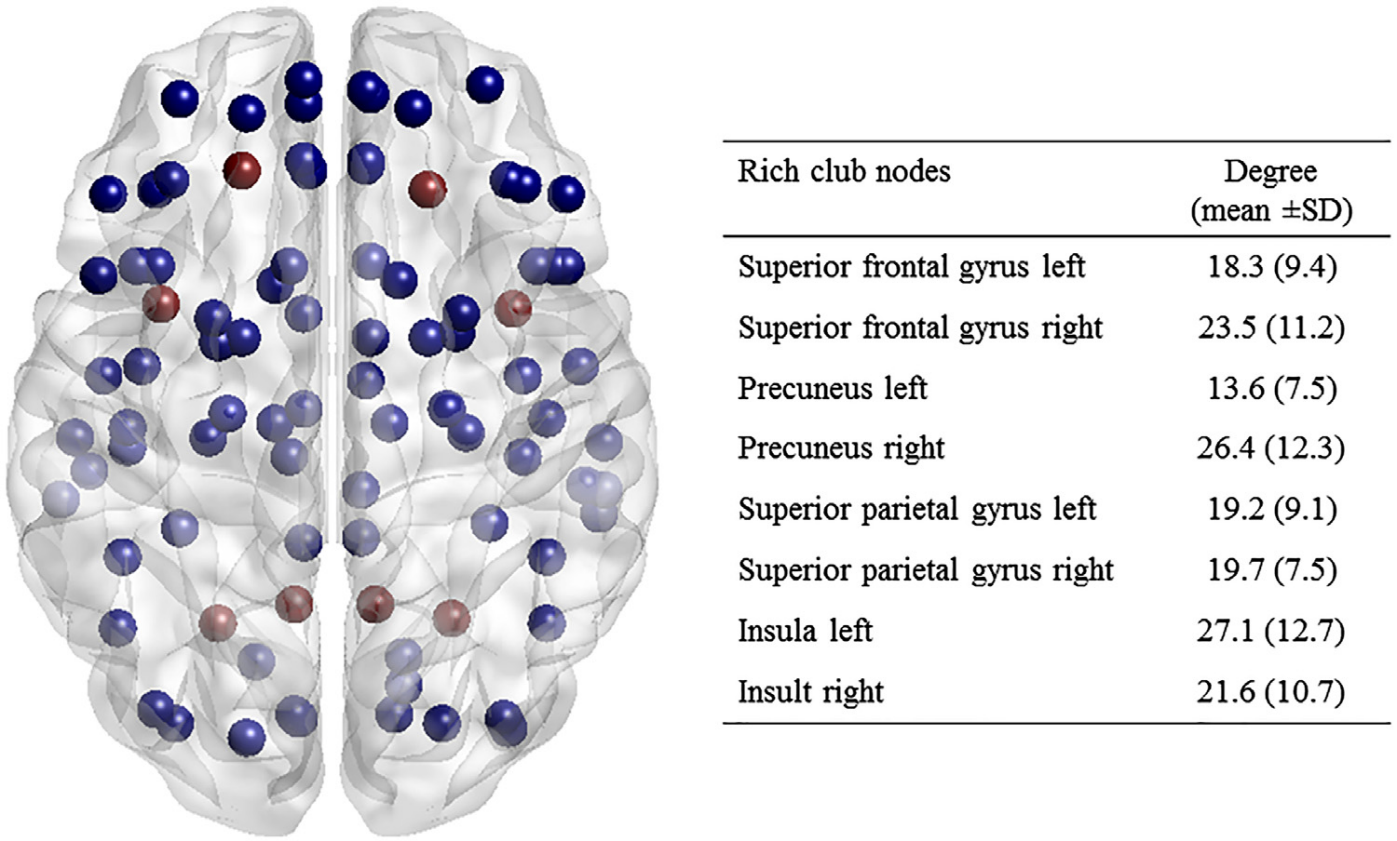
**Rich club regions** Rich club regions (red nodes) are selected based on previous literature, which include bilateral superior frontal gyrus, precuneus, superior parietal gyrus and the insula. A table is provided showing the degree of the rich club regions.

Rich club regions included the bilateral superior frontal gyrus, precuneus, superior parietal gyrus and the insula (Fig. 1). This selection of rich cub nodes was based on the literature and the selection of these nodes as rich club nodes has been validated by previous studies (Collin et al., 2014; van den Heuvel et al., 2013). The connections of the network were then classified for further analysis (van den Heuvel et al., 2012; van den Heuvel and Sporns, 2011): connections between the rich club nodes were classified as rich club connections; connections to the rich club nodes as feeder connections and connections between the non-rich club nodes as peripheral connections. The strength of these three types of connections was calculated as the average of the edge weights for that group.

### 2.12 Statistical analysis

To assess how SVD-related disturbances in structural network organization progress over time, we calculated differences in network density, network strength, global connectivity and strength of rich club, feeder and peripheral connections over time using repeated measures ANOVA. We additionally analyzed whether the changes in rich club organization differed between participants with mild versus severe WMH. Therefore, we stratified WMH severity based on median split of baseline WMH volumes. Differences between participants with mild versus severe WMH were calculated using one-way ANOVA, adjusted for age and sex.

To study the associations between conventional SVD markers (i.e. WMH and presence of lacunes and microbleeds) and structural network measures, we performed linear regression analyses, adjusted for age and sex. In addition, we assessed whether the decline in structural network organization was affected by the progression of conventional SVD markers. We therefore performed linear regression analyses using WMH progression, incident lacunes and microbleeds and difference (Δ) scores of network measures, with adjustments for age and sex.

Additionally, we aimed to relate the decline in network organization to cognitive decline. Therefore, we performed linear regression analyses, separately for decline in cognitive index, memory, psychomotor speed and executive function, adjusted for age, sex and education.

Finally, to examine whether baseline alterations in network organization are associated with dementia status at follow-up, we analyzed differences in network measures for participants with and without dementia using one-way ANOVA, adjusted for age, sex and education. To assess whether these network alterations mediated the association between conventional SVD markers with dementia, we additionally performed mediation analyses using ‘lavaan’ version 0.5–23.1097 in R (Rosseel, 2012). Using ‘lavaan’, we estimated the direct effect of baseline WMH volume on the development of dementia and the indirect effect of baseline WMH volume on the development of dementia via structural network measures, separately for global efficiency and strength of rich club, feeder and peripheral connections.

Statistical analyses were performed using R 3.5.2 (https://www.r-project.org/) and SPSS Statistics version 20.

### 2.13 Data availability statement

Data that support the findings of this study are available from the corresponding author upon request.

## 3 Results

Baseline characteristics of the study population are presented in Table 1. Mean age was 67.9 (SD 7.8) years and mean follow-up duration was 3.4 (SD 0.2) years.

**Table 1:** Characteristics of the study population.

	All (*n* = =270)	Mild WMH (*n* = =165)	Severe WMH (*n* = =105)	Dementia (*n* = =23)
Demographics				
Age, years	67.9 ± 7.8	65.3 ± 6.7	70.3 ± 7.9	78.6 ± 5.9
Male sex, number of participants (%)	162 (59.6)	82 (60.7)	78 (57.8)	14 (65.2)
MMSE score	28.4 ± 1.8	28.7 ± 1.4	28.1 ± 2.1	23.7 ± 3.7
Education, years	10.1 ± 1.5	20.3 ± 1.4	9.8 ± 1.7	8.9 ± 2.1
Vascular risk factors				
Hypertension, number of participants (%)	162 (59.6)	68 (50.4)	93 (68.9)	15 (65.2)
Diabetes, number of participants (%)	37 (13.6)	16 (11.9)	21 (15.6)	5 (21.7)
Hypercholesterolemia, number of participants (%)	124 (45.6)	55 (40.7)	69 (51.1)	13 (56.5)
Smoking, ever, number of participants (%)	194 (71.3)	93 (68.9)	99 (73.3)	18 (78.3)
Alcohol, glasses/week	3.8 ± 4.0	4.1 ± 4.4	3.6 ± 3.6	2.0 ± 1.8
Body mass index, kg/m^2^	27.8 ± 4.2	27.6 ± 4.3	28.1 ± 4.1	26.8 ± 3.3
Imaging characteristics				
Total brain volume, ml	1066.2 ± 77.7	1091.8 ± 68.5	1040.6 ± 78.3	967.8 ± 60.2
Grey matter volume, ml	610.6 ± 50.1	626.4 ± 42.4	594.7 ± 52.2	551.8 ± 30.2
White matter volume, ml	455.7 ± 44.0	465.4 ± 40.9	445.9 ± 45.1	416.0 ± 49.7
WMH volume, ml	2.8 (1.3 – 7.8)	1.3 (0.7 – 1.9)	7.7 (4.2 – 18.2)	9.3 5.3 – 27.0)
Lacunes, number of participants (%)	69 (25.4)	22 (16.3)	47 (34.8)	7 (30.4)
Microbleeds, number of participants (%)	47 (17.3)	20 (14.8)	27 (20.0)	8 (34.8)
NAWM MD, 10^−3^ mm^2^/s	0.84 ± 0.04	0.82 ± 0.02	0.86 ± 0.04	0.90 ± 0.06
NAWM FA	0.38 ± 0.02	0.39 ± 0.16	0.37 ± 0.02	0.35 ± 0.03

Data represent mean ± SD or number of participants (%). WMH volume was expressed as median (IQR). MMSE: Mini-Mental State Examination; WMH: white matter hyperintensities; NAWM: normal appearing white matter; MD: mean diffusivity; FA: fractional anisotropy.

### 3.1 Progression of rich club organization over time, by SVD severity

**Fig. 2:**
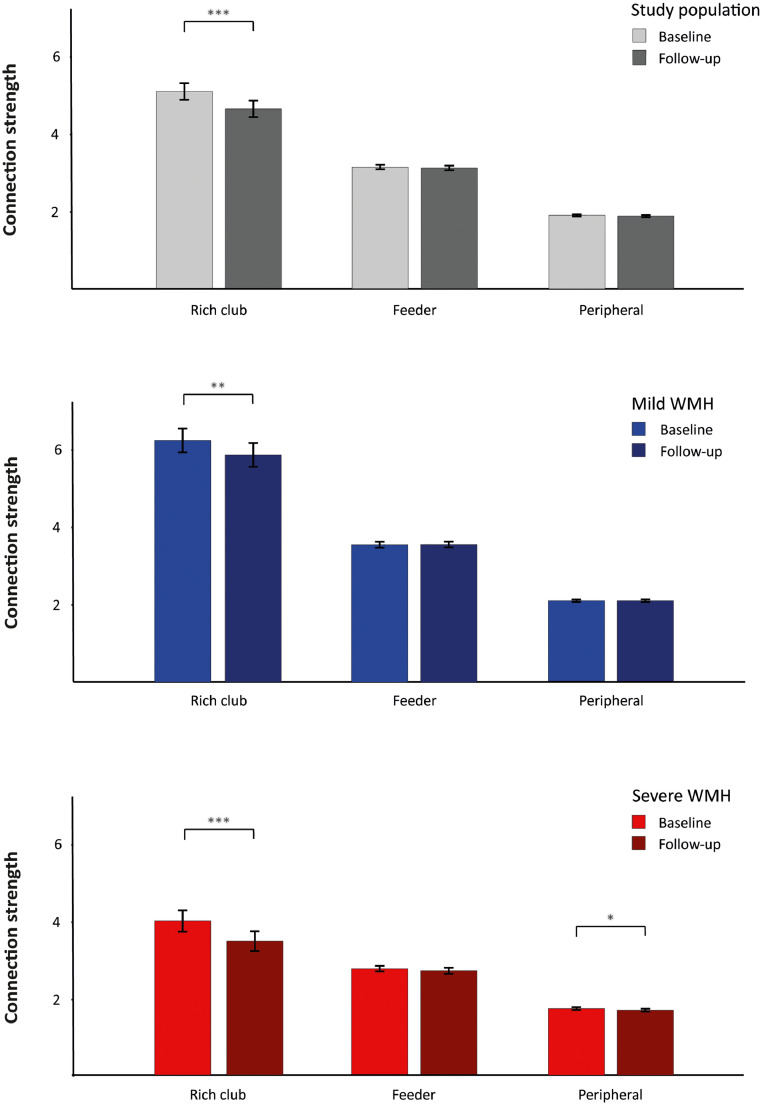
**Progression of rich club organization over time** Progression of rich club organization from baseline to follow-up. Data indicate mean connection strength ± SEM for the study population (black), and additionally for patients with mild WMH (blue) and with severe WMH (red). WMH volumes are stratified based on median split of WMH volumes in 2011. All network measures are statistically different for patients with severe versus mild WMH (*p*<0.001 for all network measures). Statistical differences between baseline and follow-up have been calculated using repeated measures ANOVA. Differences between participants with mild versus severe WMH have been calculated using one-way ANOVA, adjusted for age and sex. *p<0.05; **p<0.01; ***p<0.001.

We first assessed if and how disturbances in rich club organization progress over time. The progression of rich club organization over time is shown in Fig. 2 and Supplementary Figure 1. The strength of rich club connections declined over time (mean difference [95% confidence interval]: −0.44 [−0.63 – −0.26]; *p*<0.001), in contrast to the strength of feeder (mean difference: −0.03 [−0.073 – 0.023]; *p* = =0.303) and peripheral connections (mean difference: −0.02 [−0.040 – 0.003]; *p* = =0.094). In terms of global network measures, both network density and global efficiency declined over time. The WMH group x time interaction term was not significant, meaning that the decline in rich club connections did not differ between participants with mild and severe WMH (*p* = =0.830). In addition, the strength of peripheral connections declined in participants with severe baseline WMH, but not in participants with mild baseline WMH (Fig. 2).

### 3.2 Effects of SVD markers on changes in rich club organization

To assess whether disruptions in rich club organization are affected by the severity and progression of conventional SVD markers, we performed linear regression analyses (Table 2). The strength of rich club connections was affected by baseline WMH volume (β=−0.189; *p*<0.001) and the number of lacunes (β=−0.067, *p*<0.01). The strength of feeder and peripheral connections was affected by baseline WMH volume and by the number of lacunes and microbleeds (*p*<0.001 for all SVD markers). The progression of conventional SVD markers was not associated with changes in rich club, feeder or peripheral connections.

**Table 2:** Associations between SVD markers and rich club organization.

	Rich club strength	Feeder strength	Peripheral strength
Age, years	**−0.500^⁎⁎⁎^**	**−0.322^⁎⁎⁎^**	**−0.474^⁎⁎⁎^**
	**[−0.587, −0.414]**	**[−0.403, −0.241]**	**[−0.547, −0.400]**
Sex	**.238^⁎⁎^**	−0.038	.038
	**[.095, 0.382]**	[−0.172, 0.096]	[−0.083, 0.160]
Time, years	−0.040	.046	.025
	[−0.118, 0.038]	[−0.027, 0.120]	[−0.042, 0.091]
WMH, ml	**−0.189^⁎⁎⁎^**	**−0.336^⁎⁎⁎^**	**−0.270^⁎⁎⁎^**
	**[−0.278, −0.101]**	**[−0.419, −0.254]**	**[−0.344, −0.195]**
WMH progression, ml	−0.016	−0.041	−0.036
	[−0.094, 0.063]	[−0.115, 0.032]	[−0.102, 0.031]
Lacunes, number	**−0.067^⁎⁎^**	**−0.089^⁎⁎⁎^**	**−0.108^⁎⁎⁎^**
	**[−0.116, −0.018]**	**[−0.136, −0.043]**	**[−0.150, −0.066]**
Incident lacunes, number	.021	.002	.014
	[−0.024, 0.067]	[−0.041, 0.044]	[−0.025, 0.053]
Microbleeds, number	−0.005	**−0.033^⁎⁎⁎^**	**−0.034^⁎⁎⁎^**
	[−0.024, 0.013]	**[−0.050, −0.015]**	**[−0.049, −0.018]**
Incident microbleeds, number	.003	−0.0002	−0.003
	[−0.015, 0.021]	[−0.017, 0.017]	[−0.018, 0.013]
Total brain volume	.006	.003	.001
	[−0.010, 0.022]	[−0.013, 0.018]	[−0.013, 0.014]
Loss of total brain volume	−0.035	.003	.004
	[−0.101, 0.032]	[−0.062, 0.069]	[−0.052, 0.061]

Associations of the conventional SVD markers WMH, lacunes and microbleeds with structural network measures. WMH volumes were log-transformed because of skewedness. Data are displayed as standardized betas [95% confidence intervals], analyzed using linear regression analyses. *p<0.05; **p<0.01; ***p<0.001.

### 3.3 Effects of rich club organization on cognitive performance

The results from linear regression analyses on the associations between longitudinal changes in rich club organization and cognitive decline are shown in Table 3. The degree of reduction in global efficiency and network strength were associated with greater decline in cognitive index (global efficiency: β=0.116; *p*<0.05; network strength: β=0.147; *p*<0.01) and psychomotor speed (global efficiency: β=0.146; *p*<0.05; network strength: β=0.160; *p*<0.01). The decline in peripheral connection strength was associated with decline in cognitive index (β=0.164; *p*<0.01), psychomotor speed (β=0.151; *p*<0.05) and executive function (β=0.117; *p*<0.05) (Supplementary Figure 2). Decline in rich club or feeder connection strength was not associated with decline in cognitive performance.

**Table 3:** Rich club organization and cognitive decline.

	Δ Cognitive Index	Δ Memory	Δ Psychomotor speed	Δ Executive function
Δ Global network characteristics				
Global efficiency	.116* [.003, 0.229]	.004 [−0.115, 0.124]	.146* [.027, 0.266]	.090 [−0.029, 0.208]
Network strength	.147** [.036, 0.258]	.042 [−0.076, 0.160]	.160** [.042, 0.278]	.108 [−0.009, 0.225]
Δ Connection strength				
Rich club	.024 [−0.087, 0.136]	−0.022 [−0.140, 0.096]	.074 [−0.044, 0.194]	.000 [−0.117, 0.118]
Feeder	.059 [−0.053, 0.172]	−0.005 [−0.123, 0.113]	.115 [−0.004, 0.234]	.054 [−0.064, 0.172]
Peripheral	.164** [.054, 0.274]	.061 [−0.056, 0.178]	.151* [.034, 0.268]	.117* [.001, 0.233]

Longitudinal associations between network measures and cognitive decline. Data are displayed as standardized betas [95% confidence intervals]. Statistical differences were analyzed using linear regression analyses, adjusted for age, sex and education. *p<0.05; **p<0.01; ***p<0.001.

Mediation analyses showed that the effect of progression of WMH on the decline of cognitive index, psychomotor speeds and execution function was mediated by global efficiency (*p* = =0.029, *p* = =0.017, *p* = =0.049, respectively) and peripheral connection strength (*p* = =0.014, *p* = =0.023, *p* = =0.036, respectively), while the direct effects of the progression of WMH on the decline of cognition were not significant (Supplementary Figure 3).

### 3.4 Associations between baseline network characteristics and dementia

**Fig. 3:**
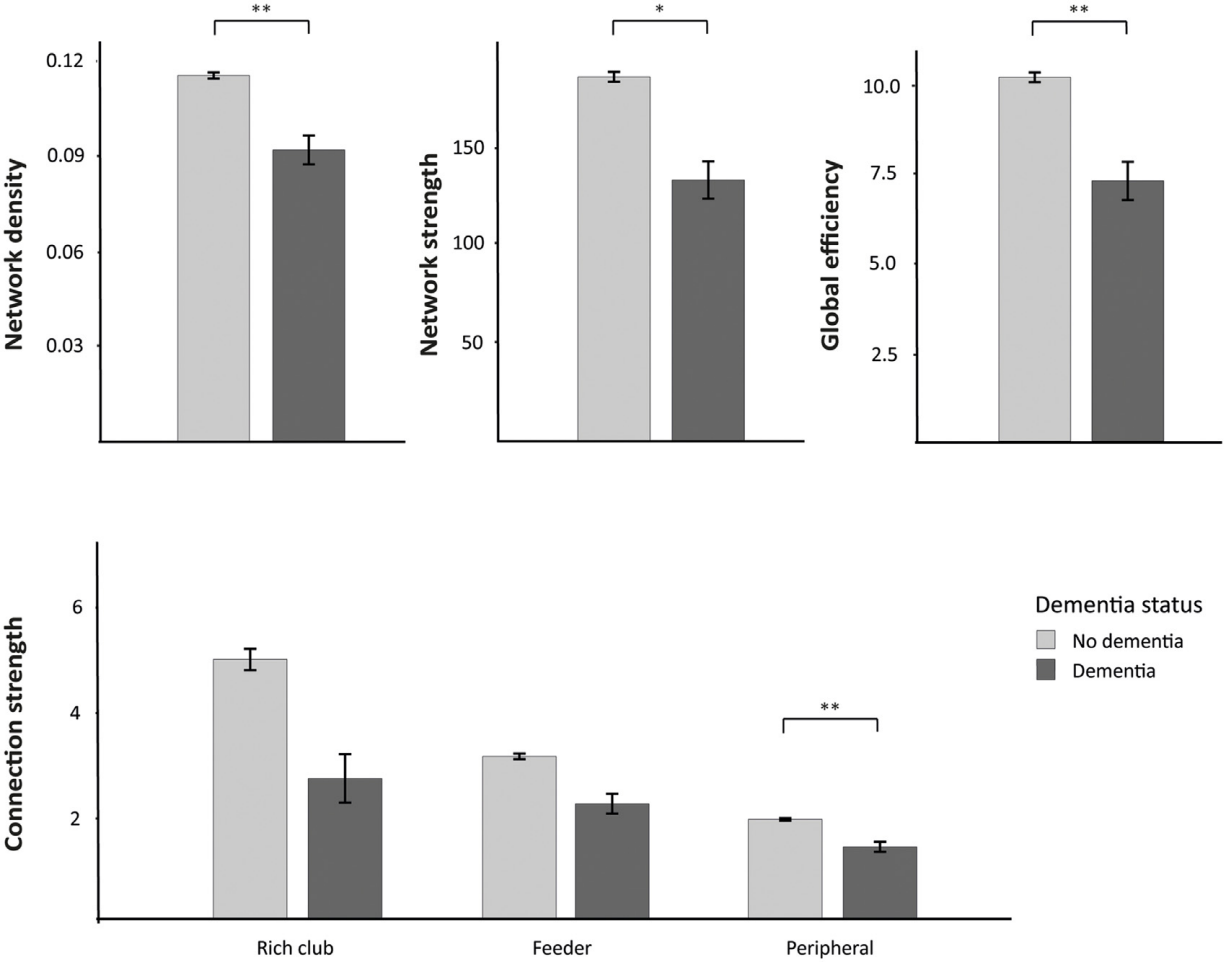
**Network characteristics at baseline stratified by dementia status at follow-up** Network characteristics at baseline, separately for participants with (dark grey, *n* = =23) and without dementia (light grey, *n* = =306). Top: The global network measures (network density, network strength and global efficiency) were reduced in participants with dementia. Bottom: Strength of rich club, feeder and peripheral connections in participants with and without dementia. Statistical differences were analyzed using one-way ANOVA, adjusted for age, sex and education. *p<0.05; **p<0.01; ***p<0.001.

**Fig. 4:**
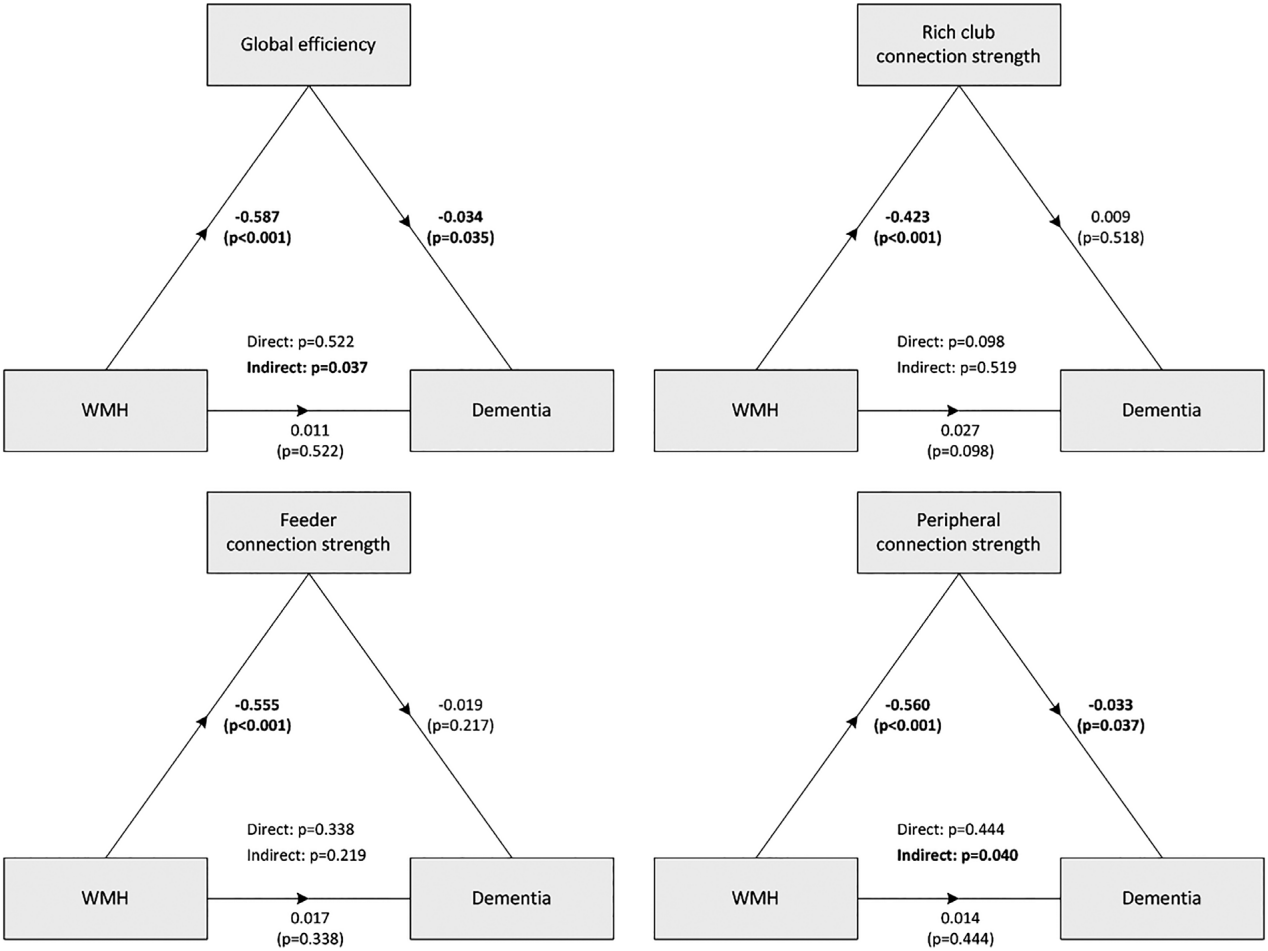
**Diagrams showing statistical mediation analyses of the relationship between WMH and dementia by structural network measures** The diagrams present standardized estimates (with p-values) for all direct associations, separately for global efficiency and strength of rich club, feeder and peripheral connections. The statistical significance of the direct and indirect paths is presented in the centre of the diagram. Dementia variable is a binary variable, diagnosed at follow-up. Analyses were performed using Lavaan, adjusted for age, sex and education. The effects of WMH on dementia were mediated by global network efficiency and the strength of peripheral connections (indirect effect), while the direct effects of WMH on dementia were not significant.

Of the 329 participants, 23 participants had been diagnosed with dementia at follow-up. We examined whether the degree of network organization at baseline examination was associated with dementia at follow-up and whether these alterations mediated the association between conventional SVD markers with dementia. Participants with dementia had, already at baseline, lower total network density (mean difference [95% confidence interval]: −0.012 [−0.019 – −0.005]; *p* = =0.001), lower network strength (mean difference: −21.0 [−38.0 – −4.0]; *p* = =0.015) and lower global efficiency (mean difference: −1.2 [−2.1 – −0.30]; *p* = =0.009) as compared with the group without dementia. Moreover, participants with dementia also showed, at baseline, lower strength of peripheral connections (Fig. 3; mean difference: −0.21 [−0.37 – −0.05]; *p* = =0.009). No differences were observed for rich club (mean difference: −0.13 [−1.2 – 1.5]; *p* = =0.848) and feeder connection strength (mean difference: −0.37 [−0.76 – 0.01]; *p* = =0.059) between participants with and without dementia. Mediation analyses showed that the effects of WMH on dementia was mediated by global network efficiency (indirect effect; *p* = =0.037) and the strength of peripheral connections (indirect effect; *p* = =0.040), while the direct effects of WMH on dementia was not significant (Fig. 4).

## 4 Discussion

In this longitudinal study, we investigated the progression of structural network connectivity and rich club organization over time in participants with SVD. SVD-related disturbances in rich club organization, specifically the strength of rich club connections, progressed significantly over 3.4 years. Declines in global network efficiency and peripheral, but not rich club or feeder, connection strength were associated with cognitive decline and dementia. The effect of WMH on dementia was causally mediated by global network efficiency and the strength of peripheral connections, suggesting an important role for global network, rather than rich club disruption in causing cognitive decline and dementia in elderly with SVD.

Our study provides evidence that SVD-related disturbances in structural networks progress over time, which in turn is related to cognitive decline. Previous cross-sectional studies have shown reductions in network global efficiency in participants with SVD that mediated the relationship between conventional MRI markers of SVD and cognitive impairment or dementia (Lawrence et al., 2014; Reijmer et al., 2015; Tuladhar et al., 2015, 2016a, 2016b). In particular, reduced connectivity was predominantly observed for rich club connections, mediating the association of WMH with processing speed and executive functioning (Tuladhar et al., 2017). However, differentiating causality from association is impossible in cross-sectional studies. Two previous longitudinal studies have reported associations between declines in global efficiency and cognitive performance in patients with cerebral amyloid angiopathy (CAA) (Reijmer et al., 2016) and in patients with severe symptomatic SVD (Lawrence et al., 2018). However, to our knowledge, no longitudinal studies have addressed the progression of disturbances in rich club organization over time in patients with SVD and its relation to subsequent cognitive decline and dementia. Our longitudinal findings support the hypothesis that conventional MRI markers of SVD (such as WMH, lacunes and microbleeds) cause cognitive decline and dementia via disruption of structural brain networks.

Several mechanisms can be hypothesized that may explain the decline of rich club connection strength over time. First, the location of incident SVD might correspond to the location of disrupted connections (i.e. WMH progression or incident lacunes or microbleeds might target specific connections and thereby disrupt white matter connections). However, we showed that, on top of baseline SVD, the progression of conventional SVD markers was not significantly associated with the strength of rich club, feeder or peripheral connections (Table 2). We therefore consider this hypothesis as less likely. An alternative explanation might be the high metabolic demand of especially the rich club nodes and connections. It has been argued that especially the rich club nodes have a high rate of metabolic activity and that the long fibers connecting the rich club nodes require higher levels of energy consumption (Bullmore and Sporns, 2009; Crossley et al., 2014). As damage in SVD is presumably caused by ischemia, the progression of small vessel damage might preferentially affect the highly metabolic rich club connections.

Disturbances in rich club organization were predominantly observed in participants with severe baseline WMH, both in rich club connections and in feeder as well as peripheral connections. These findings suggest an important role for global network function in older adults with SVD, rather than rich club disruption specifically, further supporting the notion that SVD should be considered a global rather than a focal disease (Ter Telgte et al., 2018).

Interestingly, we did not observe any associations between decline in rich club connection strength and decline in cognitive performance. While rich club connection strength was significantly associated with cognitive performance in cross-sectional analyses, cognitive decline over time was only associated with decline in peripheral connection strength, rather than with a decline in rich club or feeder connection strength. Although we are not aware of any studies investigating the preferential role of specific connections for cognitive function in elderly with SVD longitudinally, these results are in contrast to what we would expect based on findings from several cross-sectional studies reporting an important role for rich club connections in cognitive processes, specifically for psychomotor speed and executive function (Baggio et al., 2015; Tuladhar et al., 2017). There may be several explanations for not finding this association. First, it might be that the cognitive domains measured in our study reflect localized rather than global cognitive functions, for which proper network function and integration is required. However, since we observed associations between rich club connection strength and psychomotor speed and executive function – known to rely on the integration of distributed brain areas – rather than with memory performance in cross-sectional analyses, we consider this hypothesis less likely. Second, the selection of rich club nodes was based on node degree in healthy controls; although this selection has been validated in several studies (Collin et al., 2014; van den Heuvel et al., 2013) and these nodes were among the highest ranked nodes in our study population (data not shown), the rich club organization in our study population was already disrupted at baseline due to their SVD (Tuladhar et al., 2017). Possibly, the contribution of *decline* in rich club connection strength to cognitive decline, in addition to the baseline disruption of rich club connections, is limited relative to peripheral connections. Third, it might be that initial disruptions of the rich club connections are followed by secondary disruptions of the feeder or peripheral connections and that impairments in peripheral connections only lead to clinical overt symptoms after a certain threshold of structural network disruption is reached (Crossley et al., 2014). Finally, it might be that cortical structures have an important role in causing cognitive decline and dementia. Gray matter atrophy in frontal brain areas, for example, might explain executive dysfunction in participants with SVD (Gunning-Dixon and Raz, 2000). This hypothesis was supported by previous studies reporting that lower cortical thickness in frontotemporal regions was related to cognitive deficits independent of WMH (Du et al., 2005; Tuladhar et al., 2015).

Our finding that baseline measures of global efficiency and peripheral connections are impaired in participants who developed dementia is clinically relevant, because it suggests that network parameters might be useful as markers to predict cognitive decline and the risk of progression to dementia. Additionally, the finding that the effect of WMH on dementia is causally mediated by global network efficiency and the strength of peripheral connections provides additional insights into the underlying mechanisms of cognitive symptoms attributable to SVD. Altogether, these findings suggest that the structural network acts as a mediator between conventional SVD markers and cognitive outcome and may allow identification of individuals at high risk of developing dementia.

Major strengths of the study were the large cohort of individuals covering a wide range of the SVD spectrum, the detailed phenotyping of the patients, including the diagnosis of dementia according to a standardized approach in all patients and the availability of longitudinal neuroimaging data obtained from the same scanner without upgrade or change over the full data collection period. However, several methodological issues should also be considered. First, the identification of structural networks was based on DTI acquired at 1.5 Tesla with relatively few diffusion directions and deterministic streamlining using tensor reconstruction models, limiting the identification of long-distance fibers and the reconstruction of white matter tracts in a complex white matter architecture due to noise and partial volume effects (Zalesky and Fornito, 2009). Although high-resolution imaging and more advanced tractography methods are required to provide more detailed information about the white matter architecture, the consistency of our findings on impaired global efficiency in SVD and its relation with cognitive performance with other studies (Lawrence et al., 2014; Reijmer et al., 2015; Tuladhar et al., 2016a) indicate that our network analyses in participants with SVD are reliable. Second, it might as well be that our observed changes in rich club organization and cognitive performance over time are not solely attributable to SVD, but also to the effects of normal aging or to other pathologies such as neurodegeneration or Alzheimer's disease, or interaction with these pathologies. However, the associations between conventional SVD markers with measures of rich club organization and the causal mediations of the associations between WMH and dementia via structural network properties indicate that the disruption of structural networks is important in explaining cognitive decline in elderly with SVD.

## 5 Conclusions

The strength of rich club connections significantly declined over time, whereas declines in global efficiency and peripheral connection strength were associated with cognitive decline and dementia. The effect of WMH on dementia was mediated by global network efficiency and the strength of peripheral connections, suggesting an important role for global, rather than rich club network disruption in causing cognitive decline and dementia in elderly with SVD.

## Disclosures

Prof. Dr. de Leeuw is supported by a clinical established investigator grant of the Dutch Heart Foundation (grant number 2014 T060), and by a VIDI innovational grant from The Netherlands Organisation for Health Research and Development (ZonMw grant 016.126.351). Dr. Tuladhar is supported by a junior staff member grant of the Dutch Heart Foundation (grant number 2016 T044).

## Appendix Supplementary materials

Image, application 1
