# Supplementary material for: Longitudinal changes in rich club organization and cognition in cerebral small vessel disease
Source: Neuroimage Clin. 2019 Oct 22;24:102048. doi: 10.1016/j.nicl.2019.102048 (PMC6978216; doi:10.1016/j.nicl.2019.102048)
Supplement: Supplementary file 1 [file mmc1.docx]

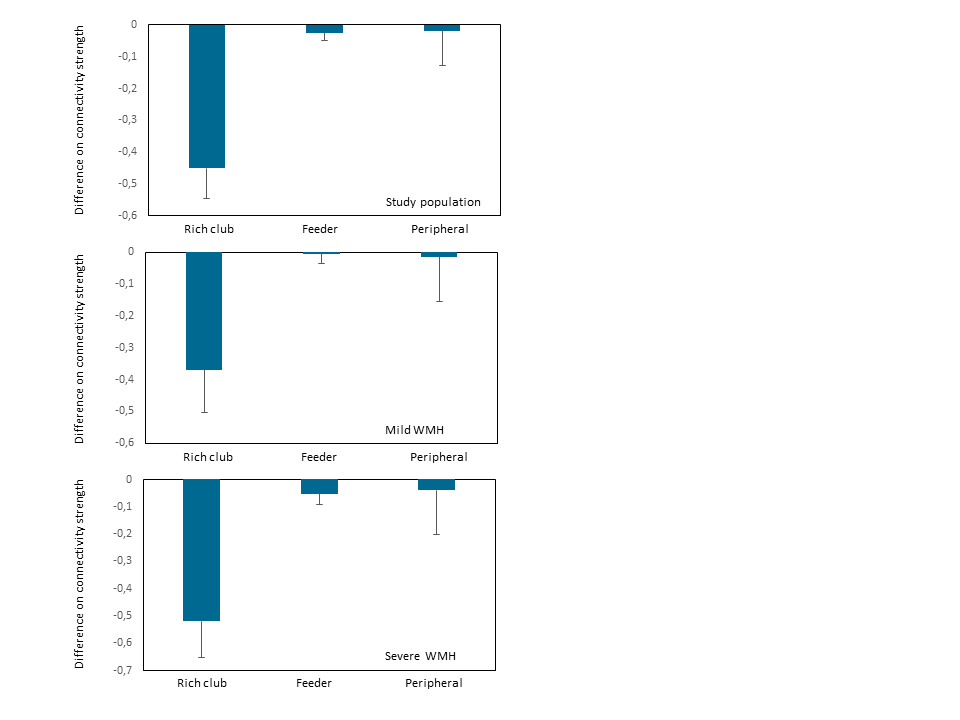


**Supplementary Figure 1.** Pairwise differences of the connectivity strength among the three groups. Negative values indicate decline over time.


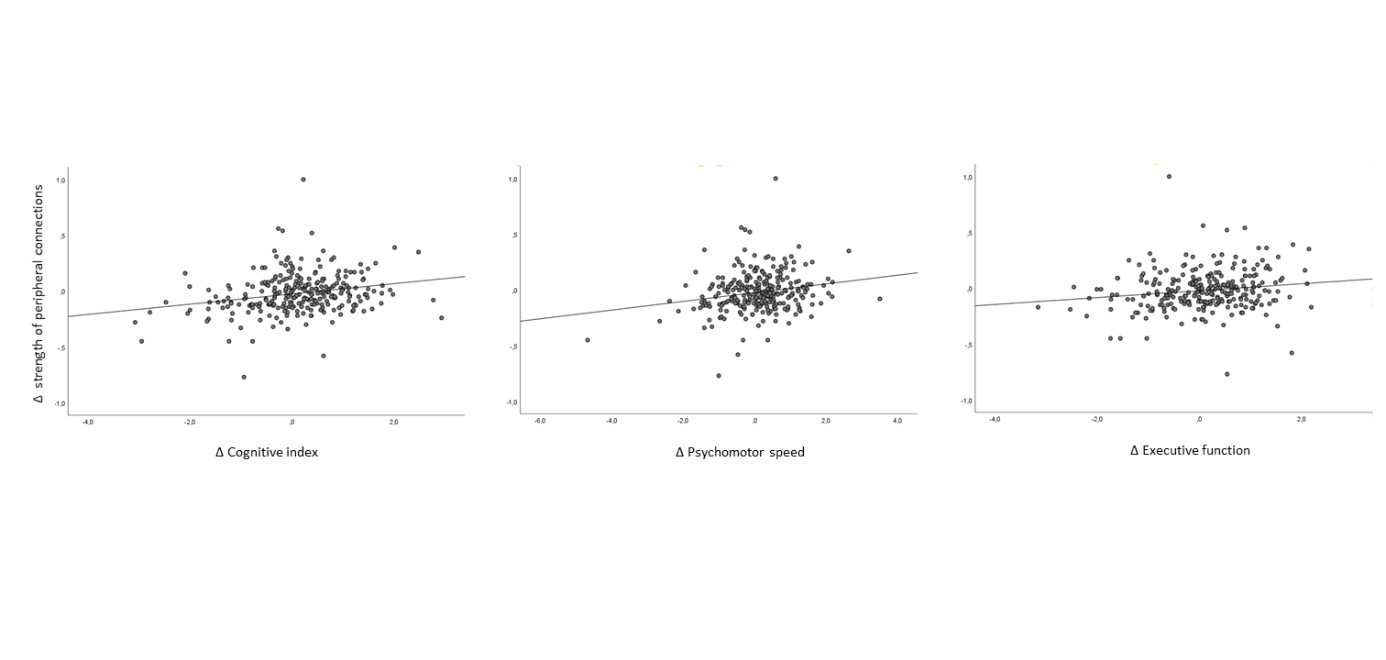


**Supplementary Figure 2.** Correlations between change in connectivity strength of the peripheral connections and change in cognitions for the significant associations (see Table 2). Negative values indicate decline over time.


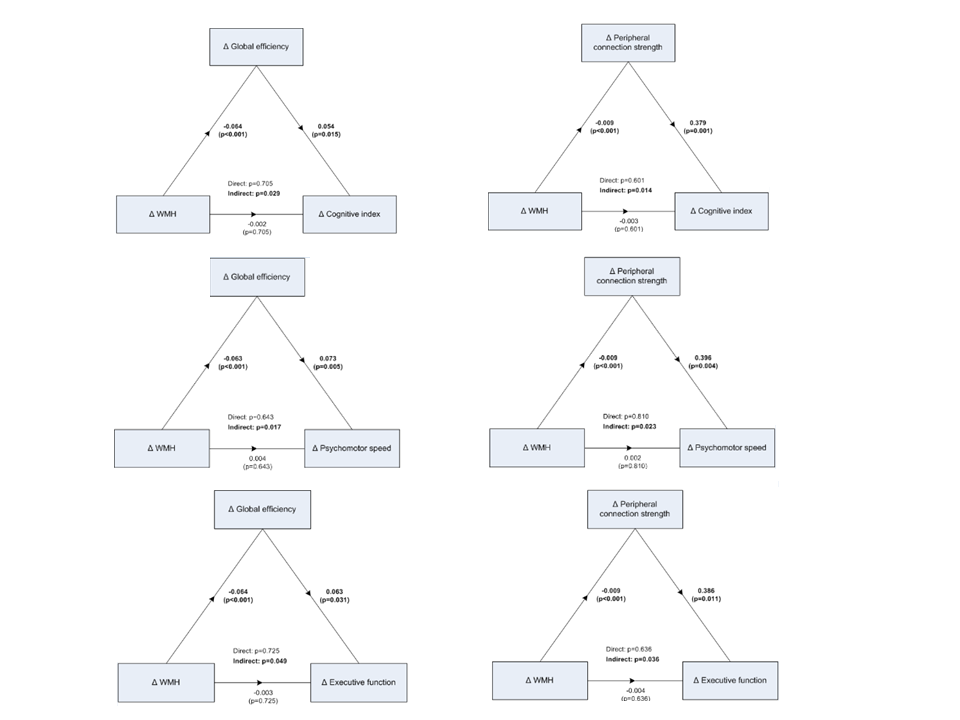


**Supplementary Figure 3.** **Diagrams showing statistical mediation analyses of the relationship between progression WMH and decline in cognition by decline structural network measures.**

The diagrams present standardized estimates (with p-values) for all direct associations, separately for global efficiency and strength of peripheral connections. The statistical significance of the direct and indirect paths is presented in the centre of the diagram. Analyses were performed using Lavaan, adjusted for age, sex and education. Mediation analyses showed
